# Metabolism of a synthetic compared with a natural therapeutic pulmonary surfactant in adult mice[S]

**DOI:** 10.1194/jlr.M085431

**Published:** 2018-08-14

**Authors:** Jens Madsen, Madhuriben H. Panchal, Rose-Marie A. Mackay, Mercedes Echaide, Grielof Koster, Giancarlo Aquino, Nicola Pelizzi, Jesus Perez-Gil, Fabrizio Salomone, Howard W. Clark, Anthony D. Postle

**Affiliations:** Child Health, Clinical and Experimental Sciences,* Faculty of Medicine, University of Southampton, Southampton, United Kingdom; Department of Biochemistry and Molecular Biology,† Faculty of Biology, Hospital 12 de Octubre Research Institute, Complutense University, Madrid, Spain; National Institute for Health Research,§ Biomedical Research Centre, University Hospital Southampton, Southampton, United Kingdom; Chiesi Farmaceutici SpA,** Parma, Italy

**Keywords:** phosphatidylcholine synthesis, molecular species, stable isotopes, acyl remodeling, mass spectrometry

## Abstract

Secreted pulmonary surfactant phosphatidylcholine (PC) has a complex intra-alveolar metabolism that involves uptake and recycling by alveolar type II epithelial cells, catabolism by alveolar macrophages, and loss up the bronchial tree. We compared the in vivo metabolism of animal-derived poractant alfa (Curosurf) and a synthetic surfactant (CHF5633) in adult male C57BL/6 mice. The mice were dosed intranasally with either surfactant (80 mg/kg body weight) containing universally ^13^C-labeled dipalmitoyl PC (DPPC) as a tracer. The loss of [U^13^C]DPPC from bronchoalveolar lavage and lung parenchyma, together with the incorporation of ^13^C-hydrolysis fragments into new PC molecular species, was monitored by electrospray ionization tandem mass spectrometry. The catabolism of CHF5633 was considerably delayed compared with poractant alfa, the hydrolysis products of which were cleared more rapidly. There was no selective resynthesis of DPPC and, strikingly, acyl remodeling resulted in preferential synthesis of polyunsaturated PC species. In conclusion, both surfactants were metabolized by similar pathways, but the slower catabolism of CHF5633 resulted in longer residence time in the airways and enhanced recycling of its hydrolysis products into new PC species.

The maintenance of optimal pulmonary surfactant function and metabolism is critical in minimizing surface tension forces within the lungs and preventing alveolar collapse at the end of expiration. Primary insufficiency of the immature lungs of preterm infants is the principal cause of neonatal respiratory distress syndrome (1). Secondary surfactant dysfunction also contributes to the severe lung pathology of adult patients ventilated for acute respiratory distress syndrome due to a combination of increased concentrations of inhibitory edema proteins (2–4), phospholipase-mediated hydrolysis of surfactant phospholipids (5), oxidant and antioxidant imbalance (6), and damage to the alveolar type II (ATII) epithelial cell responsible for surfactant synthesis and secretion (7).

Phospholipids represent the major surface-active components of surfactant, with cholesterol and surfactant proteins B and C regulating their fluidity (8) and rapid adsorption to the alveolar air-liquid interface (9, 10). Phospholipid composition is dominated by phosphatidylcholine (PC), with a high content of the disaturated species dipalmitoylphosphatidylcholine (DPPC) compared with typical mammalian cell membranes (11). This elevated DPPC, the major surface-active agent, is maintained by a complex intra-alveolar metabolism. PC is synthesized on the endoplasmic reticulum of the ATII cell with a lower content of DPPC (12, 13) and is then subjected to selective ABCA3-mediated transport into anhydrous lamellar storage organelles (14, 15) combined with a process of acyl remodeling catalyzed by sequential phospholipase A_2_ and lysophosphatidylcholine (lysoPC) acyltransferase activities (13, 16). Surfactant enriched in DPPC is then secreted into the alveolar lining fluid by exocytosis of lamellar bodies, followed by rapid adsorption to the air-liquid interface. Surfactant is subsequently catabolized by ATII cell endocytosis (17), metabolism by alveolar macrophages (18), and loss up the bronchial tree (19). A proportion of the surfactants taken up by ATII cells is subsequently recycled into lamellar bodies for resecretion into the alveolus (20, 21). Studies in rabbits indicate that surfactant recycling is more active in neonatal compared with adult animals (22), leading to slower apparent alveolar turnover and increased half-life.

While these metabolic pathways have been well-defined by elegant studies that monitored the incorporation into PC of substrates labeled with radioactive isotopes (12, 13), there is a paucity of information available regarding their regulation in molecular terms, which are the biologically relevant molecules defined by their combination of esterified fatty acids. Many studies have relied on the quantification of total disaturated PC species after OsO_4_ oxidation of unsaturated species (23), but this analytical approach provides minimal information at the level of individual PC molecular species. Second, analyzing radioactive-labeled PC species is laborious and time-consuming (13), and the use of radioactivity is precluded in clinical studies.

Our group has developed methodologies to probe the metabolism of surfactant molecular species in greater detail (24, 25). These protocols monitor the incorporation of stable isotope-labeled substrates into PC molecular species, which are then resolved by electrospray ionization MS/MS. The incorporation of deuterated methyl-D_9_-choline is common to the de novo synthesis of all PC molecular species by the CDP-choline pathway, and we have established mechanisms of acyl remodeling of surfactant PC synthesis by animal models (25) and adult volunteers and acute respiratory distress syndrome patients (26, 27).

In this study, we aimed to explore the differences between the catabolism, turnover, and metabolism of two exogenous pulmonary surfactants: CHF5633, which is a new synthetic surfactant produced by Chiesi Farmaceutici (Parma, Italy) currently under phase-II clinical investigations for neonatal respiratory distress syndrome treatment (28), and poractant alfa (Curosurf; Chiesi Farmaceutici), an animal-derived surfactant preparation commonly used in clinical practice (29). CHF5633 contains DPPC and 1-palmitoyl-2-oleoylglycero-3-phospho-1-glycerol (1:1) and incorporates analogs of both surfactant proteins B and C. This synthetic preparation has already been subjected to several translational in vivo acute studies that showed an efficacy profile similar to poractant alfa (30, 31). Nevertheless, while poractant alfa metabolism has been investigated in animal studies (32), CHF5633 metabolism needs to be explored in order to evaluate whether its synthetic origin and molecular composition differentiate its long-term fate following administration compared with animal-derived surfactants. Indeed, CHF5633 does not contain any polyunsaturated phospholipids, and its surfactant protein B and C analogs do not contain any amino acids (such as methionine, which is on the contrary present in the natural surfactant proteins) that is sensitive to oxidation (30). In this study, we investigated the catabolism and metabolism of poractant alfa and CHF5633 containing as a tracer DPPC, where all 40 carbon atoms were replaced by the stable carbon-13 isotope [U^13^C]DPPC. The use of [U^13^C]DPPC has several significant advantages compared with partially labeled DPPC. First, it is very unlikely that hydrolysis products will be recycled back into fully labeled DPPC, and consequently the loss of [U^13^C]DPPC will represent catabolism in the absence of recycling. Second, as all hydrolysis products will also be fully ^13^C-labeled, the fate of their incorporation into all potential metabolic products can be readily assessed. The study was undertaken by administering [U^13^C]DPPC-labeled surfactants to adult mice. We also studied endogenous surfactant production and turnover in adult mice by giving them methyl-D_9_-choline as a precursor for the synthesis of lung PC to establish any potential for the inhibition of endogenous PC synthesis by exogenous surfactants.

## MATERIAL AND METHODS

### Preparation of labeled surfactants

#### Poractant alfa.

[U^13^C]DPPC (48.4 mg) was dissolved in 4 ml dichloromethane and transferred to a 500 ml round-bottom Quickfit flask to which total lipid extracts of four vials of poractant alfa (960 mg total weight), prepared using dichloromethane and methanol, were added. The organic solvent was removed by rotary evaporation under vacuum for 30 min at 40°C to provide a thin film of dried lipid, followed by purging with nitrogen gas to remove any residual solvent. PBS (10 ml) was then added to the flask, and the dried lipid extract was solvated by rotary evaporation without a vacuum for 30 min at 40°C. The resultant labeled surfactant emulsion was then stored in 1 ml aliquots at 4°C at a final DPPC concentration of 34 mg/ml.

#### CHF5633.

[U^13^C]DPPC was formulated into the fully synthetic surfactant during the production phase, replacing an equivalent amount of unlabeled DPPC (Chiesi Farmaceutici). The functional performance of both U^13^C-labeled surfactant preparations was judged to be fully comparable to their corresponding nonlabeled counterparts as assayed in the captive bubble surfactometer (31, 33) (see supplemental Fig. S1). Labeled and unlabeled preparations had comparable ability to adsorb rapidly into the air-water interface to form films capable of reproducibly reaching surface tensions <5 mN/m upon repetitive compression-expansion cycling. Dynamic compression-expansion isotherms of poractant alfa and CHF5633 had distinctive features that were fully mirrored by each corresponding labeled preparation.

### Animal labeling and sample preparation procedure

All animal procedures were approved internally by the University of Southampton Animal Welfare and Ethical Review Body and externally by the Home Office Animals in Science Regulation Unit. Male C57BL/6 wild-type mice aged 8–12 weeks were used for this study. The mice were bred in-house and kept under a normal 12 h dark/light cycle with free access to pelleted food and water. All animals in this study received appropriate care according to the criteria outlined in Kilkenny et al. (34). Each mouse was intranasally instilled with 50 µl (4 mg; equivalent to 200 mg/kg body weight) of either poractant alfa or the synthetic surfactant CHF5633 containing [U^13^C]DPPC as described previously and successfully found to deliver material to the alveoli of the lung (35). At the same time, each mouse also received a 100 µl intraperitoneal injection of methyl-D_9_-choline chloride (10 mg/ml in water). After labeling, the mice were euthanized by carbon dioxide asphyxia at 0, 1.5, 3, 6, 12, 18, 24, 48, 72, and 96 h (n = 10–21 mice per time point/group). Bronchoalveolar lavage was performed in situ with 4 × 0.9 ml PBS, and the recovered bronchoalveolar lavage fluid (BALF) aliquots were combined. BALF was centrifuged at 300 *g* for 10 min at 4°C to pellet cells, and the supernatants were then transferred to new vials and stored at −80°C until extraction. Lung parenchyma was quickly dissected from the main bronchi, placed in cryotubes, and snap-frozen in liquid nitrogen. Right and left lung lobes were stored separately at −80°C until further analysis.

### Phospholipid extraction

Lavaged right lung lobes were weighed and homogenized in 1.6 ml of 0.9% saline using a Heidolph Silent Crusher S. Total lipid extraction was performed by the Bligh and Dyer method on 800 µl aliquots of lung homogenates or BALF supernatants after the addition of a dimyristoyl PC (10 nmol) internal standard to each sample (36). Dichloromethane (2 ml), methanol (2 ml), and water (1 ml) were added to each sample, mixed well to allow for the formation of a biphasic mixture, and then centrifuged at 1,500 *g* for 10 min at 20°C. The dichloromethane-rich lower phase was recovered, dried under a stream of nitrogen gas, and stored at −20°C until analysis by MS.

### MS analysis

MS analysis was performed on a Waters XEVO TQ-MS instrument using electrospray ionization. Dried samples were dissolved in a 1 ml mixture of methanol-dichloromethane-concentrated ammonium acetate (300 mM) in water (66:30:4 [v/v]). The sample solution was infused into the instrument without chromatography using the loop injection method. Different diagnostic precursor scans were performed to detect the different head group species. The use of different MS approaches was successfully applied for the characterization of phospholipid classes of poractant alfa (37). In this study, the various diagnostic MS/MS scans used for characterizing PC metabolism are summarized in **Table 1**. Unlabeled PC and newly synthesized PC labeled with [D_9_]choline were calculated from precursor ion scans of phosphorylcholine fragment ions at *m/z* 184 and *m/z* 193, respectively. Precursor ion scans of *m/z* 189 detected the PC species containing five labeled ^13^C atoms in their choline head group. The various neutral loss scans all detected DPPC species with a variety of labeled components. Neutral loss scans of 551 and 586 detected unlabeled DPPC and [U^13^C]DPPC, respectively, while the ions at 737 and 771 were their respective M+3^+^ and M-3^+^ isotopomers. All other neutral loss scans detected DPPC species incorporating the various ^13^C-metabolic products of [U^13^C]DPPC hydrolysis.

**TABLE 1.** Diagnostic MS/MS scans for the analysis of PC metabolism

Diagnostic MS/MS Scans for Choline Head Groups			
MS/MS Mode	Lipids	Range (*m/z*)	Characterization
Precursor *m/z* 184	PC	400–900	PC species containing phosphorylcholine
Precursor *m/z* 189	[5^13^C]PC	400–900	PC species containing [5^13^C]choline
Precursor *m/z* 193	[D_9_]PC	400–900	PC species containing [D_9_]choline

Diagnostic MS/MS Scans for DPPC with Different Labeled Diacylglycerol Components				
MS/MS Mode	Lipids	Range (*m/z*)	Ion (*m/z*)	Characterization
Neutral loss 551	DPPC	732–745	734.5	Unlabeled DPPC
Neutral loss 554	[3^13^C]DPPC	735–748	737.5	DPPC—[3^13^C]glycerol or DPPC M+3^+^
Neutral loss 567	[16^13^C]DPPC	748–761	750.8	DPPC—one 16^13^C palmitate
Neutral loss 570	[19^13^C]DPPC	751–764	753.5	DPPC—one 16^13^C palmitate + [3^13^C]glycerol
	[24^13^C]DPPC		758.5	DPPC—[24^13^C]lysoPC16:0 + [16^12^C]palmitate
Neutral loss 583	[32^13^C]DPPC	764–777	766.5	DPPC—two 16^13^C palmitates
	[37^13^C]DPPC		771.5	[U^13^C]DPPC M-3^+^
Neutral loss 586	[U^13^C]DPPC	767–780	774.5	[U^13^C]DPPC

Diagnostic MS/MS Precursor Ion Scans in Negative Ionization			
MS/MS Mode	Lipids	Range	Characterization
Precursor 255	PC	400–900	PC species containing unlabeled palmitate
Precursor 271	[16^13^C]PC	400–900	PC species containing [16^13^C]palmitate

Spectra were processed using a visual basic macro program developed in-house. Initially, MS spectra were smoothed, baseline-subtracted, and exported to individual Excel files. These files were then imported into the macro program and corrected for ^12^C- or ^13^C-isotopic effects. Enrichment values for individual molecular species were calculated from the percentage ratio of the corrected abundances of the stable isotope-labeled isotopomer to the sum of all isotopomers. For [U^13^C]DPPC, as an example, the calculation would be as follows:

$${\text{U}}^{13}\text{C-DPPC enrichment}\left(\%\right)=\frac{\text{Abundance}\left\{{\text{U}}^{13}\text{C-DPPC}\right\}\times 100}{\text{Abundance}\left\{{\text{DPPC}+\text{U}}^{13}\text{C-DPPC}+{\text{D}}_{9}\text{-DPPC}\right\}}$$

These calculated enrichment values were then normalized using the enrichments of [U^13^C]DPPC in the labeled CHF5633 and poractant alfa preparations according to the following formula:

$$\text{normalized data}=\frac{\left(\text{measured enrichment of U}13\text{C}-\text{DPPC}\right)\times 100}{\text{Enrichment of label material given}}.$$

### Lipid analysis of labeled surfactants

The composition and enrichment of [U^13^C]DPPC surfactants were also determined by electrospray ionization MS using the same phospholipid extraction protocol as mentioned above for samples. Enrichment values were determined using the same precursor ions, giving values for two batches of poractant alfa of 4.54% and 7.55% and one for CHF5633 of 2.87%.

## RESULTS

**Fig. 1.**
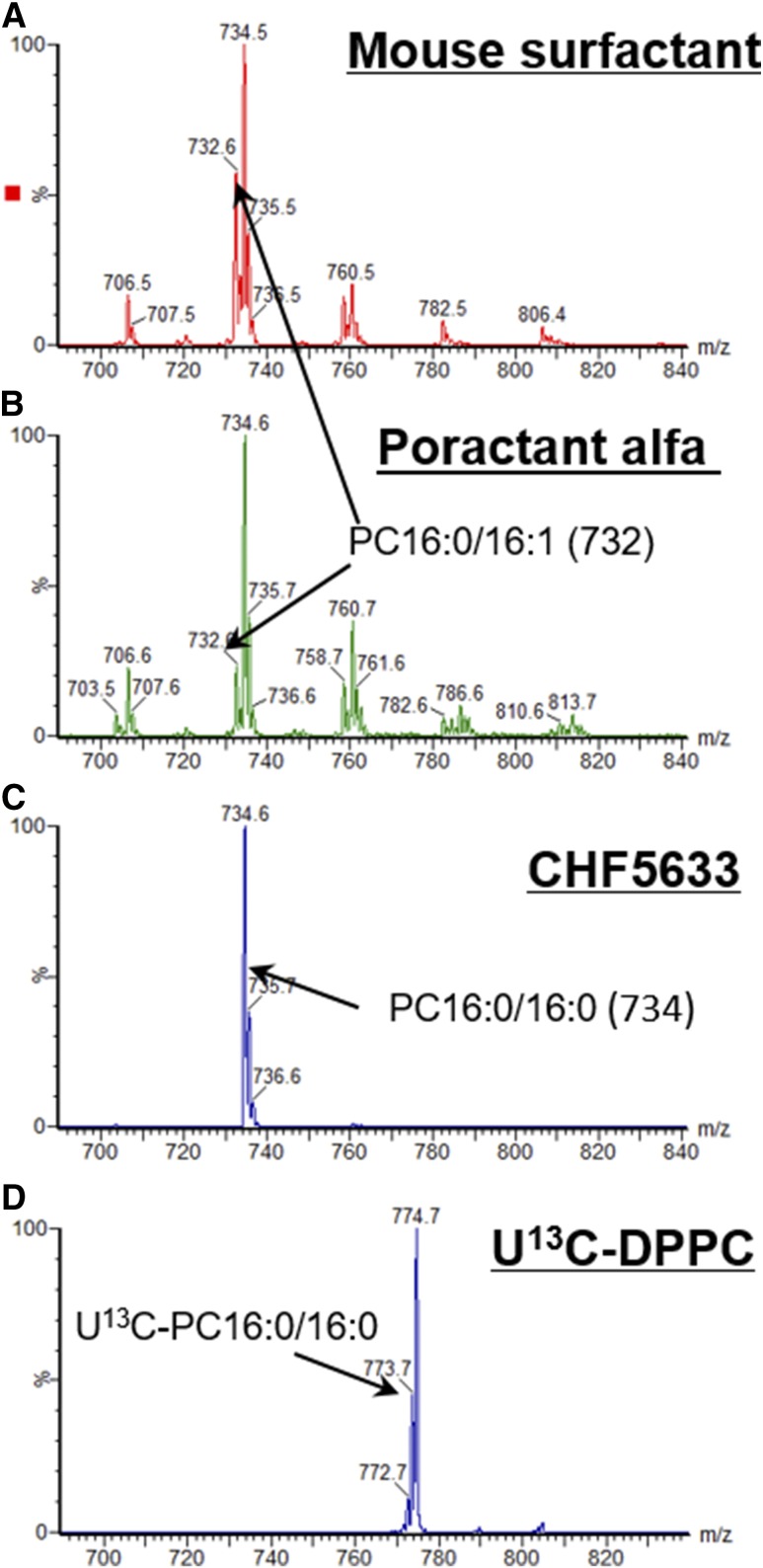
PC molecular species analysis of the mouse lung and exogenous surfactants. Compositions of mouse surfactant (A), poractant alfa (B), and CHD5633 (C) detailed by precursor scanning of the phosphocholine ion fragment at *m/z* 184. Precursor scanning of the [5^13^C]phosphocholine head group confirmed that [U^13^C]DPPC was >99% isotopically labeled (D).

Diagnostic precursor scans of the phosphocholine ion fragment readily distinguished ^13^C-labeled PC from unlabeled PC. Precursor ion scans of *m/z* 184 (P184) detected the unlabeled PC composition of the mouse surfactant, poractant alfa, and CHF5633 (**Fig. 1A**–C). MS/MS fragmentation of [U^13^C]DPPC generated an ion product of *m/z* 189 that contained five ^13^C atoms and, consequently, a precursor ion scan of *m/z* 189 (P189) detected [U^13^C]DPPC (Fig. 1D). The P189 scan was then used to quantify exogenous surfactant catabolism.

**Fig. 2.**
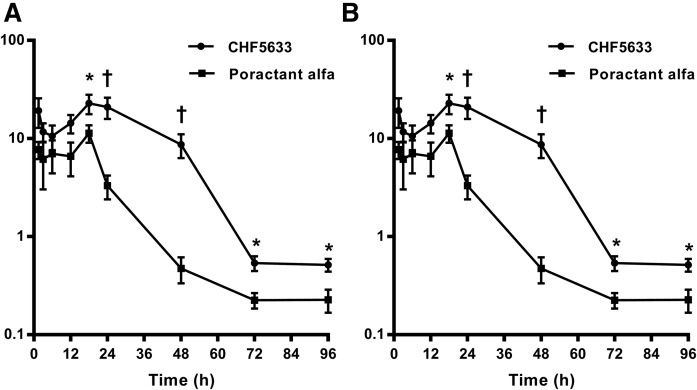
Time dependence of exogenous surfactant DPPC enrichment in mouse lung BALF (A) and in the lavaged lung (B). Results were calculated by normalizing [U^13^C]DPPC enrichments for the ^13^C enrichment of DPPC in the administered surfactant. Results are presented as means ± SEMs. *P < 0.05, ^†^*P* < 0.01, and ^‡^*P* <0.001, unpaired *t*-test.

While the mouse surfactant (Fig. 1A) and poractant alfa (Fig. 1B) contain similar molecular species of PC, they have different compositions, with the mouse surfactant containing double the percentage distribution of the monounsaturated species PC32:1 (PC16:0/16:1; *m/z* 732) and CHF5633 containing only PC32:0 (PC16:0/16:0; *m/z* 734). As shown in supplemental Fig. S2, these differences in composition are important for providing an independent verification of the turnover of the labeled surfactants. As expected from a fully labeled molecule, the isotopomer pattern for [U^13^C]DPPC was M-1, -2, -3 instead of M+1, +2, +3, demonstrating isotopomers with one, two, or three ^12^C atoms. An analysis of this isotopomer pattern indicated that [U^13^C]DPPC was isotopically ∼99% enriched with the label (**Fig. 2D**), but it also contained minor amounts of labeled contaminants (e.g., *m/z* 804). These contaminants, however, did not significantly impair the characterization of any PC species that contained ^13^C-labeled moieties derived from the metabolism of [U^13^C]DPPC (see below). Compositions of phosphatidylglycerol (precursor scan *m/z* 153) and phosphatidylinositol (precursor scan *m/z* 241) were very similar for the mouse surfactant and poractant alfa and, as expected, CHF5633 contained only PG16:0/18:1 (*m/z* 747) (results not shown).

### Validation of labeled surfactant preparations

We had two potential concerns about the use of this heavily labeled DPPC as a tracer of surfactant metabolism. First, would the presence of the labeled material significantly impair the physical surface tension-reducing properties of either surfactant and, second, would exogenous labeled and unlabeled surfactants be metabolized at equivalent rates? Captive bubble surfactometer analysis (supplemental Fig. S1) confirmed that both poractant alfa and CHF5633 retained entirely comparable surface properties after formulation with their nonlabeled counterparts. Initial adsorption isotherms are typically different, as exhibited by CHF5633 and poractant alfa, and the ones shown in supplemental Fig. S1 are representative of such a difference. Poractant alfa always adsorbs to the interface very rapidly, reaching close to equilibrium surface tensions in a few seconds. There are no significant differences as a consequence of the reconstitution of an isotopically labeled version of poractant alfa with respect to the original formulation. In contrast, CHF5633 typically exhibits a somewhat heterogeneous behavior at initial adsorption, with a sharp decay in surface tension to the equilibrium values occurring at variable times (from a few to tens of seconds) after injection. This is the reason why initial adsorption isotherms of CHF5633 exhibit frequently large standard deviations, as a consequence of averaging isotherms with the surface tension decay occurring at different times. Still, the behavior in terms of initial adsorption is again not significantly different when comparing CHF5633 with its isotopically labeled version. In the two surfactants, CHF5633 and poractant alfa, postexpansion adsorption is always extremely efficient, and the behavior of the original and labeled versions is fully comparable with respect to its interfacial spreading and compression-expansion dynamics.

A variety of MS analysis strongly argues against any selective metabolism of the labeled DPPC. For this, we first calculated the concentration of exogenous surfactants in BALF from all mice at all time points using the [U^13^C]DPPC enrichment. These values were then correlated with separate estimates of exogenous surfactant concentration in BALF based on the PC compositional differences shown in Fig. 1 (supplemental Fig. S2). Comparable values for exogenous surfactant concentration were provided by both approaches, demonstrating similar clearance rates of labeled and unlabeled components of exogenous surfactants.

### Catabolism of [U^13^C]DPPC surfactants by mouse lungs

Surfactant preparations were delivered to the mouse lungs by intranasal administration. While this method avoided the need for invasive procedures and ensured animal survival for the required number of days, it resulted in very variable delivery, which was apparent even at the earlier time points. This variation is shown in supplemental Fig. S3, which details enrichments of [U^13^C]DPPC in BALF and lung tissue, expressed as a percentage of total DPPC, for both poractant alfa and CHF5633. The grouped enrichment results are summarized for BALF (Fig. 2A) and lung tissue (Fig. 2B), with between 10 and 21 mice per data point to minimize the variation effects. Two major conclusions are evident from these results. First, the catabolism of [^13^C]DPPC was low for the first 24 h for both surfactants and, second, virtually no intact [U^13^C]DPPC remained for either surfactant after 72 h in either BALF or lung tissue. The results also suggest that CHF5633 was metabolized more slowly than poractant alfa between 24 and 72 h; the enrichment of exogenous surfactant was statistically significantly greater for CHF5633 than poractant alfa at 24, 48, 72, and 96 h in BALF (Fig. 2A) and at 18, 24, 48, and 72 h in lung tissue (Fig. 2B). One consequence of the apparent longer retention in lung tissue of CHF5633 compared with poractant alfa was a greater extent of metabolism and remodeling of the synthetic surfactant (summarized below).

### De novo synthesis of endogenous PC by lung tissue

**Fig. 3.**
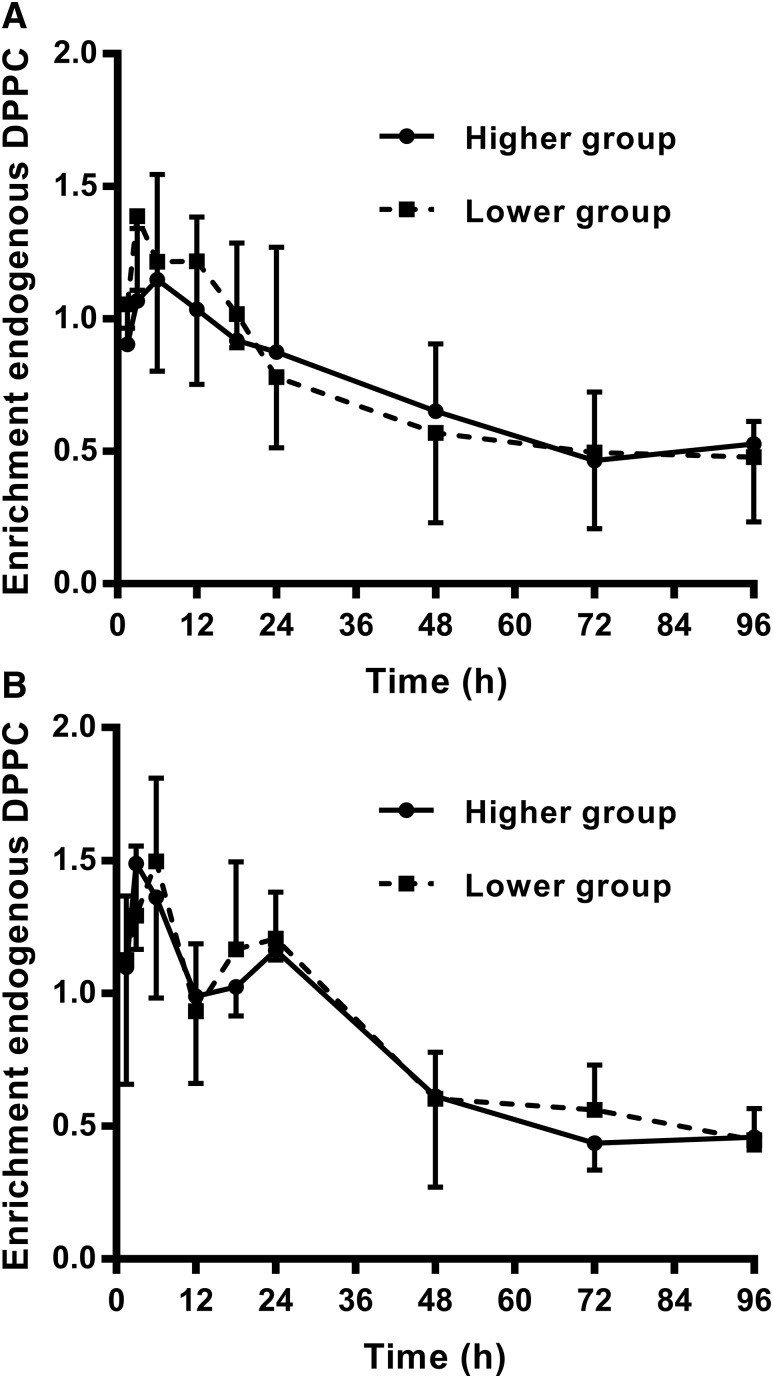
Effect of exogenous surfactant on the synthesis of endogenous DPPC by the lungs of mice administered CHF5633 (A) or poractant alfa (B). The enrichment of [D_9_]choline in endogenous DPPC was determined after correcting for the concentration of exogenous DPPC. Mice were divided at each time point into two equal groups based on the variation of exogenous surfactant enrichment in BALF. Any inhibition of PC synthesis by exogenous surfactant would have been seen by lower [D_9_]choline DPPC enrichment in the group of mice that received a higher dose of surfactant.

One important question is the extent to which increased surfactant concentration in the lungs after exogenous administration might affect de novo PC synthesis. The kinetics of endogenous PC synthesis in mouse lungs by the CDP-choline pathway were determined from the incorporation of methyl-D_9_-choline. Results from the CHF5633 and poractant alfa surfactant-treated mice were generally similar in timescales, although enrichment values tended to be lower than in the control data from previous studies (13). The incorporation of methyl-D_9_-choline into endogenous PC was calculated after correcting for the concentration of exogenous PC, which was determined from the [U^13^]CDPPC enrichment of the sample. The considerable variation in the exogenous surfactant concentration was then used to divide individual mice at each time point into two equal groups based on greater or lesser enrichment of [U^13^C]DPPC in BALF. The rationale was that if exogenous surfactants inhibited endogenous surfactant synthesis, then [D_9_]choline enrichment into DPPC, calculated relative to endogenous DPPC, would be lower in the group with higher exogenous surfactant content. As is apparent in **Fig. 3A** and B, there was no such difference for either CHF5633- or poractant alfa-treated mice, indicating negligible inhibition of endogenous surfactant PC synthesis after the administration of exogenous surfactants.

**Fig. 4.**
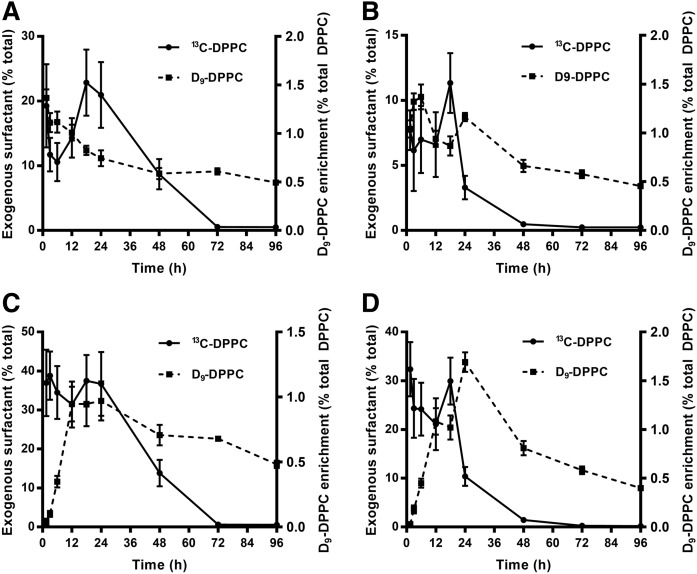
Time dependence of [U^13^C]DPPC and [D_9_]DPPC enrichment in the lungs (A, B) and BALF (C, D) for mice administered CHF5633 (A, C) or poractant alfa (B, D). Results for [U^13^C]DPPC were normalized for stable isotope enrichments of administered surfactants to enable a comparison between CHF5633 and poractant alfa. Results are presented as means ± SEMs.

A comparison of catabolism results demonstrated that exogenous surfactant [U^13^]DPPC clearance decreased significantly more rapidly than endogenously synthesized DPPC (**Fig. 4**). The plots in Fig. 4 are for the enrichment of [U^13^C]DPPC and [D_9_]choline DPPC for lung tissue (Fig. 4A, B) and BALF supernatant (Fig. 4C, D) for both CHF5633 (Fig. 4A, C) and poractant alfa (Fig. 4B, D). While [U^13^C]DPPC from both CHF5633 and poractant alfa surfactants had essentially disappeared by 72 h, [D_9_]choline DPPC enrichments remained high even at 96 h. This result was due to the different labeling patterns between the two stable isotope-labeled DPPC species. Once [U^13^C]DPPC is metabolized, it is unlikely for intact uniformly labeled [^13^C]DPPC to be resynthesized. By comparison, the enrichment of the [D_9_]choline label also reflects resynthesis and recycling of lung surfactant PC. Consequently, the difference between clearances of exogenously and endogenously labeled DPPC provides an indication of the extent of surfactant recycling in vivo.

### Metabolism of exogenous and endogenous surfactant PC molecular species

**Fig. 5.**
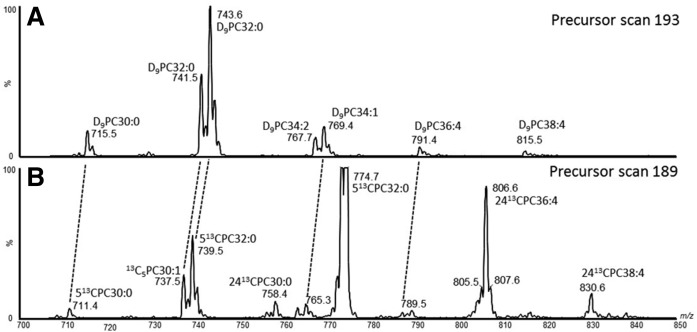
Precursor ion scanning of labeled BALF PC. A: Precursor scan of *m/z* 193 detailing the incorporation of methyl-D_9_-choline into PC species 9 amu greater than unlabeled PC. B: Precursor ion scan of *m/z* 189 showing the incorporation into PC species of [5^13^C]choline derived from [U^13^C]DPPC. The incorporation of [5^13^C]choline by the CDP-choline pathway is given by odd-numbered species 4 amu lower than in A (e.g., *m/z* 739.5 vs. 743.6). The addition of an unlabeled acyl chain to [24^13^C]lysoPC16:0 is indicated by even-numbered species (e.g., *m/z* 806.6 and 830.6). These spectra are from a mouse 48 h after being administered CHF5633 and methyl-D_9_-choline.

The precursor ion scan of *m/z* 193 illustrates the pattern of incorporation of methyl-D_9_-choline into individual molecular species of BALF PC, in this example from a mouse 24 h after CHF5633 administration (**Fig. 5A**). [D_9_]choline PC species are 9 amu greater than corresponding unlabeled PC species (Fig. 1A). The spectrum in Fig. 5B details the corresponding precursor ion scan of *m/z* 189, which displays all PC species that incorporate [5^13^C]choline derived by either phospholipase C or D hydrolysis of [U^13^C]DPPC. Consequently, in addition to quantifying [U^13^C]DPPC, this P189 scan contains two series of PC metabolic products that contained this [5^13^C]choline head group. The first series (*m/z* 711,4, 737.5, 739.5, and 787.7) represents [5^13^C]choline complexed with endogenous unlabeled diacylglycerol species. The second series (*m/z* 758.4, 806.6, and 830.6) represents PC species derived by acyl remodeling of unlabeled acyl CoAs with [24^13^C]lysoPC16:0. Comparison with the upper spectrum (Fig. 5A) indicates that [5^13^C]choline and [D_9_]choline were incorporated into the same PC species with comparable abundance distributions but displaced by 4 amu. By contrast, the 24^13^C-labeled PC had a very different distribution, with much higher labeling of polyunsaturated PC species at *m/z* 806.6 ([24^13^C]PC16:0/20:4) and *m/z* 830.6 ([24^13^C]PC16:0/22:6) than *m/z* 758.4 ([24^13^C]PC16:0/16:0).

### Recycling of [5^13^C]choline into lung PC molecular species by the CDP-choline pathway

**Fig. 6.**
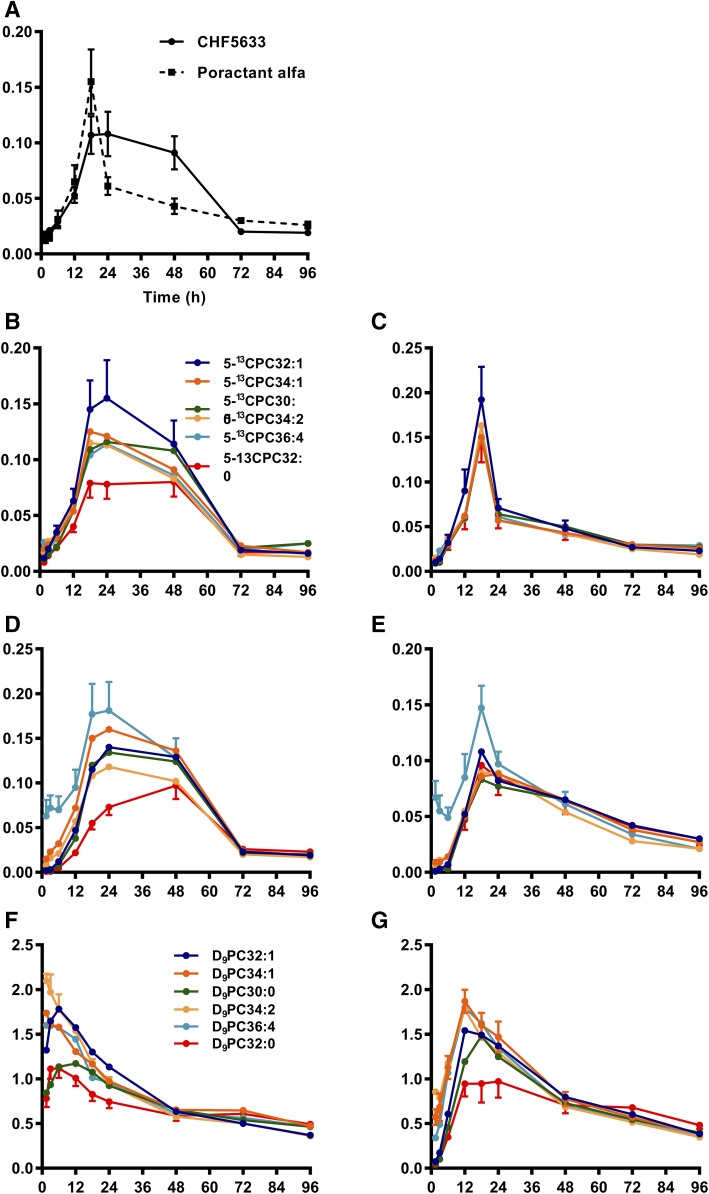
Incorporation of labeled choline into PC molecular species of the mouse lung (A, B, C, and F) and BALF (D, E, and G). Incorporation of [5^13^C]choline derived from the hydrolysis of [U^13^C]DPPC is shown for the enrichments of total [5^13^C]PC species in the lungs (A), individual [5^13^C]PC species in the lungs from CHF5633- (B) and poractant alfa-treated mice (C), and individual [5^13^C]PC species in BALF from CHD5633- (D) and poractant alfa-treated mice (E). Results were normalized for the enrichment of [U^13^C]DPPC in administered surfactant to enable a direct comparison between CHF5633- and poractant alfa-treated mice. The incorporation of methyl-D_9_-choline is shown in F and G for the lungs and BALF for CHF5633-treated mice. Results are presented as means ± SEMs.

The incorporation of the [5^13^C]choline group into [5^13^C]PC by the CDP-choline pathway is detailed in **Fig. 6**. The incorporation into total lung PC was more prolonged for CHF5633-treated mice compared with poractant alfa-treated mice (Fig. 6A), a result that is consistent with the greater residence time of intact [U^13^C]DPPC in these mice (Fig. 2). We interpret this finding to a slower generation of hydrolysis products in the CHF5633 group, giving more time for their recycling into PC species. The incorporation of [5^13^C]choline into selected individual molecular species of PC is shown for lung tissue (Fig. 6B, C) and BALF (Fig. 6D, E). Results are clearer for CHF5633, but similar trends were apparent for both surfactant treatments. There was no preferential incorporation of labeling into DPPC, with maximal enrichments for polyunsaturated (PC36:4) and monounsaturated (PC34:1) species. The lower enrichment of [5^13^C]DPPC compared with the other PC species for CHF5633-treated mice was due to the increased fractional concentration of unlabeled DPPC in these mice. These results show clearly that while there is remodeling of the head group of the surfactant PC, this is not specific for the resynthesis of DPPC. It is important to note that the incorporation of labeling into DPPC is greatest in absolute terms due to the higher concentration of DPPC in the lungs, but the enrichment results clearly show this is not preferentially enhanced compared with the other PC species. It is interesting to note that the timescale of these incorporations was considerably delayed compared with that of methyl-D_9_-choline (Fig. 3), presumably reflecting a relatively slow release of [^13^C]choline by the hydrolysis of [U^13^C]DPPC.

For comparison, the pattern of incorporation of methyl-D_9_-choline into the same molecular species of lung tissue (Fig. 6F) and BALF (Fig. 6G) is shown for CHF5633-treated mice. Similar results were obtained for poractant alfa-treated mice (results not shown). The similarity of their incorporation patterns strongly supports the conclusion that [5^13^C]choline enters the substrate pool for the de novo synthesis of PC by the CDP-choline pathway rather than being incorporated by head group exchange.

### Acyl remodeling of lung PC

**Fig. 7.**
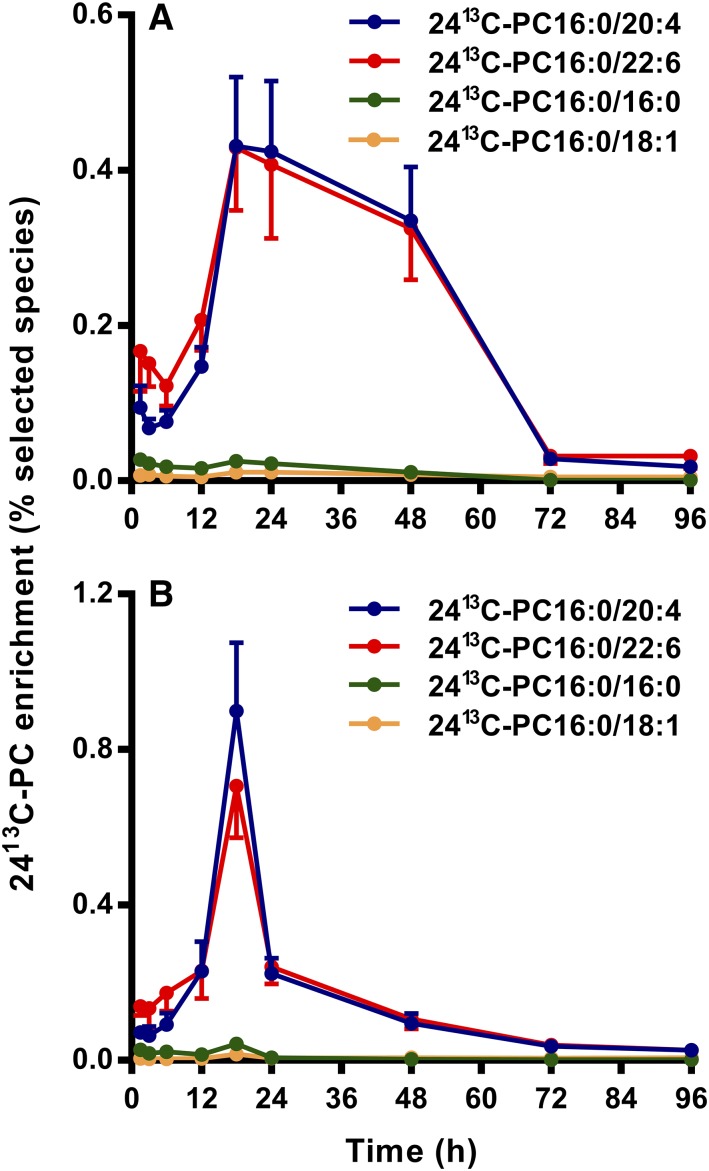
Selectivity of acyl remodeling of [24^13^C]lysoPC16:0 in mouse lungs for CHF5633 (A) and poractant alfa (B). [24^13^C]lysoPC16:0 was generated by the action of phospholipase A_2_ on [U^13^C]DPPC, followed by reacylation with an unlabeled [^12^C]acyl residue. Results are shown for percentage label enrichments as means ± SEMs for [24^13^C]PC16:0/20:4 and [24^13^C]PC16:0/22:6 and only as errors for [24^13^C]PC16:0/16:0 and [24^13^C]PC16:0/18:1. All other PC species were too small to display.

PC molecules containing 24^13^C atoms are generated when one labeled palmitate is removed from [U^13^C]DPPC by action of a phospholipase A and replaced by an unlabeled fatty acid. Results for the 24^13^C enrichment of selected PC species in the lung show clearly that there was no preferential resynthesis of DPPC from [24^13^C]lysoPC16:0 for either CHF5633 (**Fig. 7A**) or poractant alfa (Fig. 7B). Instead, there was a consistent and progressive incorporation of labeling into two polyunsaturated PC species: PC16:0/20:4 (*m/z* 806) and PC16:0/22:6 (*m/z* 830). Similar enrichment patterns were apparent for the same analysis in BALF supernatants (results not shown). Given the retention of [^13^C]palmitoyl on the [24^13^C]lysoPC16:0 intermediate, we can be confident in the assignments of these incorporated polyunsaturated fatty acids. Further analysis indicated there was no accumulation of [24^13^C]lysoPC16:0 in lung tissue, as the enrichment of [24^13^C]lysoPC16:0 was lower than that of either [24^13^C]PC16:0/20:4 or [24^13^C]PC16:0/22:6 (results not shown). This suggests a close integration of the processes of PC hydrolysis and acyl remodeling. The low enrichment of [24^13^C]PC16:0/16:0 (*m/z* 758) was unexpected because acyl remodeling of newly synthesized unsaturated PC species to DPPC is critical for the synthesis and packaging of surfactants. This discrepancy suggests that lysoPC derived from the hydrolysis of exogenous surfactant was not in equilibrium in the intact lungs with the pool of lysoPC used for the synthesis of endogenous surfactant DPPC. However, the site for the reacylation of [24^13^C]lysoPC16:0 was not clear and may well have occurred in cells other than ATII epithelial cells, such as bronchial epithelial cells or alveolar macrophages, both of which are relatively enriched in arachidonoyl PC species compared with the lung parenchyma (38).

### Recycling of surfactant [U^13^C]DPPC

**Fig. 8.**
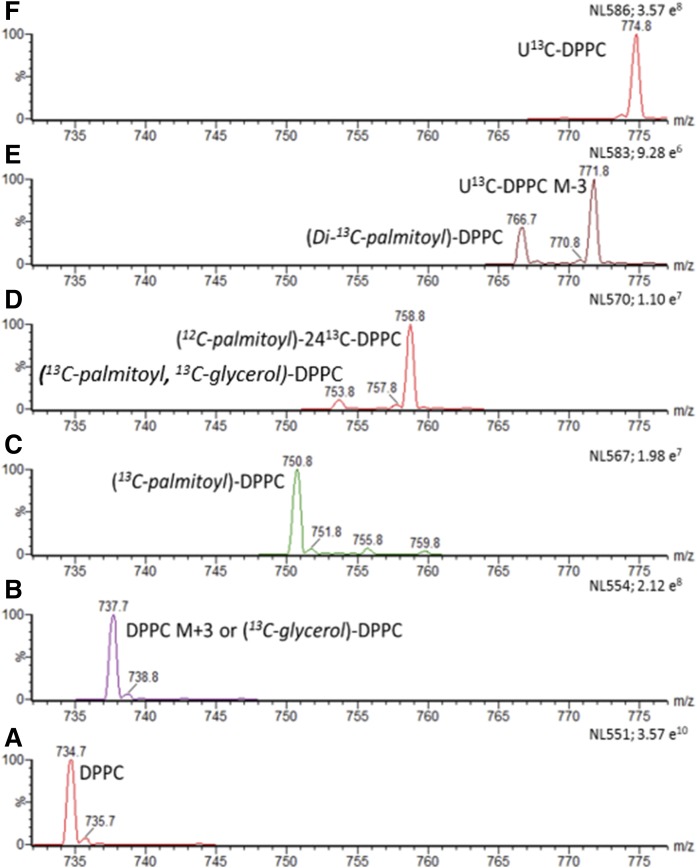
Recycling of [U^13^C]DPPC. Neutral loss scans illustrating the metabolic routes whereby [U^13^C]DPPC is metabolized and labeled fragments are recycled into BALF DPPC. The scans were derived from a mouse that received [U^13^C]DPPC-labeled CHF5633 for 24 h. A: Unlabeled DPPC. B: M+3 isotopomer of DPPC and potentially [^13^C]glycerol-labeled DPPC. C: Incorporation of one [^13^C]palmitoyl group into DPPC. D: Reacylation of [24^13^C]lysoPC16:0 with an unlabeled [^12^C]palmitoyl group. E: Combination of two [^13^C]palmitoyl groups with unlabeled [^12^C]glycerophosphocholine (*m/z* 766.7) and M-3 isotopomer of [U^13^C]DPPC (*m/z* 771.8). F: [U^13^C]DPPC.

**Fig. 9.**
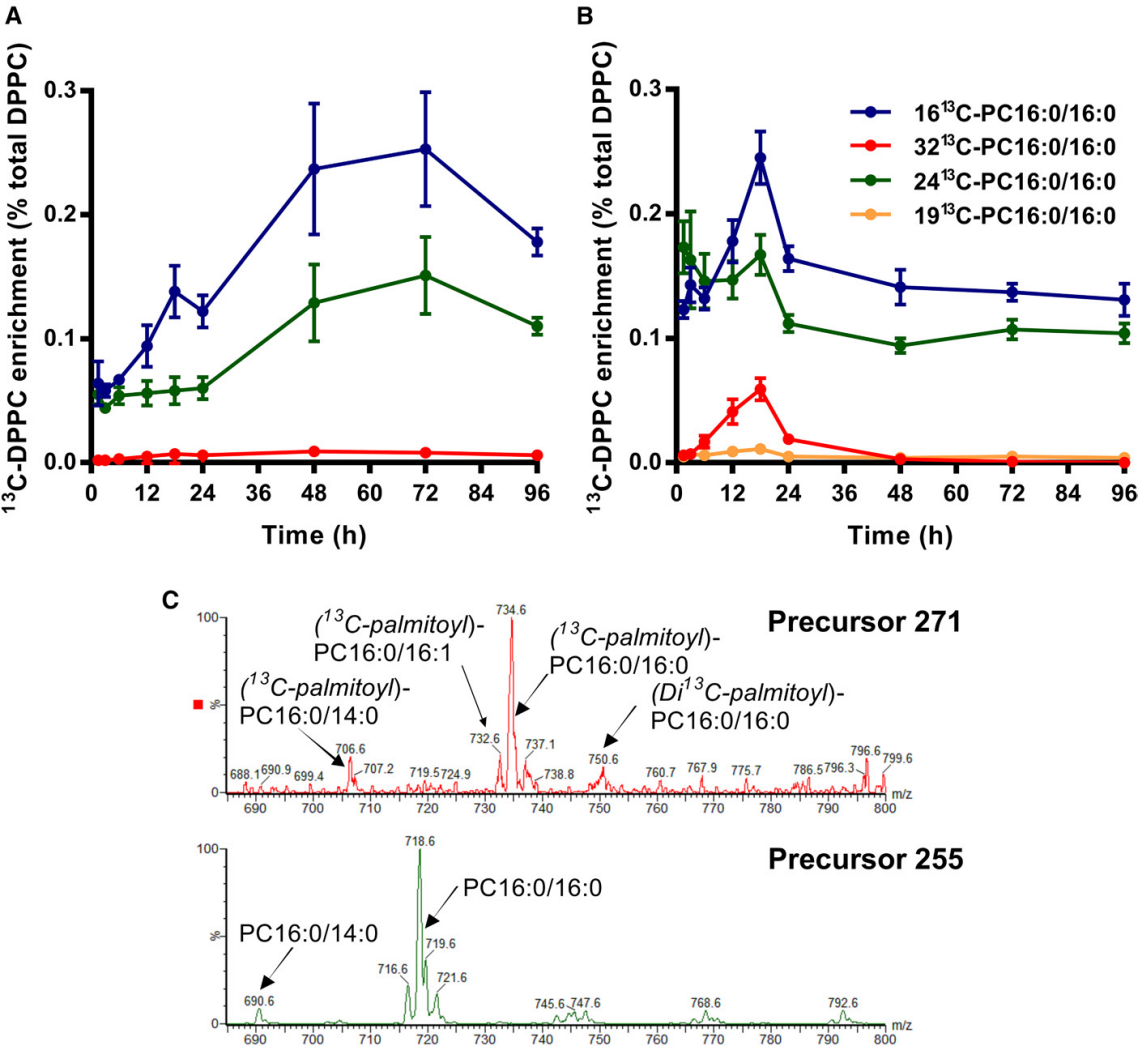
Recycling of [^13^C]palmitate into BALF DPPC by mouse lungs in vivo. Neutral loss scans of *m/z* 567 and 583 detected the incorporation of [^13^C]palmitate derived from [U^13^C]DPPC back into, respectively, ([^13^C]palmitoyl)-DPPC ([16^13^C]PC16:0/16:0; *m/z*750) and (di-^13^C-palmitoyl)-DPPC ([32^13^C]PC16:0/16:0; *m/z* 766) in BALF from mice administered either CHF 5633 (A) or poractant alfa (B). The corresponding neutral loss scan of *m/z* 570 details the acylation of [24^13^C]lysoPC16:0 with unlabeled palmitate to form ([^12^C]palmitoyl)-[24^13^C]DPPC ([24^13^C]PC16:0/16:0; *m/z* 758) and DPPC containing both labeled palmitate and glycerol ([^13^C]palmitoyl and [^13^C]glycerol-DPPC; *m/z* 753). Results are presented as means ± SEMs. C: Precursor scans of palmitoyl (*m/z* 255) and [16^13^C]palmitoyl (*m/z* 271) into PC species in negative ionization. PC species were detected as M-16^−^ ions. This spectrum was at 72 h after surfactant administration, which is why no ion peak for [U^13^C]DPPC-16^−^ was observable at *m/z* 758.6.

The potential routes for recycling of the stable isotope label from [U^13^C]DPPC back into DPPC were explored by a series of scans of the diacyl neutral loss fragments (**Fig. 8**). For instance, NL551 showed unlabeled DPPC (Fig. 8A), while NL586 showed [U^13^C]DPPC (Fig. 8F). The low incorporation of unlabeled palmitate into [24^13^C]PC16:0/16:0 (Fig. 7) was apparent for NL570 (Fig. 8D). Less certain assignments can be given to the other identified ions (Fig. 8B, C, E), as similar results could be given by endogenous PC species. However, time-dependent analysis (**Fig. 9**) suggests that incorporated [^13^C]palmitoyl represents at least a component of these neutral loss scans. The higher initial signal from the poractant alfa-treated mice was due to those mice receiving surfactants with a greater label enrichment. The enrichments of *m/z* 750 (NL567), 753 (NL570), and 766 (NL583) increased with time, supporting the conclusion that these represented incorporations of [^13^C]palmitoyl into [^13^C]palmitoyl-DPPC and di-^13^C*-*palmitoyl-DPPC, respectively. Independent support for the incorporation of [^13^C]palmitate into PC species was provided by precursor ion scans of *m/z* 255 ([^12^C]palmitate) and *m/z* 271 ([^13^C]palmitate) in negative ionization, when PC species were detected as M-16^−^ ions (Fig. 9C). Fig. 9C details the incorporation of ([^13^C]palmitate) into ([^13^C]palmitoyl)-PC16:0/14:0, ([^13^C]palmitoyl)-PC16:0/16:1, ([^13^C]palmitoyl)-PC16:0/16:0, and (di-^13^C-palmitoyl)*-*PC16:0/16:0.

## DISCUSSION

The results of this study indicate that the DPPC component of the synthetic surfactant CHF5633 was cleared from the lungs more slowly than the poractant alfa porcine-derived surfactant. This is demonstrated by the longer duration of enrichment of [U^13^C]DPPC from CHF5633 both in BALF (Fig. 2A) and postlavage lung tissue (Fig. 2B) and by the more prolonged generation of PC species labeled with ^13^C products of [U^13^C]DPPC hydrolysis (Figs. 6, 7, 9). The duration of recycling into PC of the [5^13^C]choline fragment (Fig. 6A, B) and the [24^13^C]lysoPC16:0 fragment (Fig. 7A, B) clearly shows that, although both surfactants were metabolized by equivalent pathways, poractant alfa and its metabolic products were cleared more rapidly than CH5633. The reason for this difference is not clear but may partially be related to the slightly higher loading of lungs with DPPC achieved by CHF5633 compared with poractant alfa. Whereas both surfactants were administered at the same total phospholipid concentration and dose (200 mg/kg), the delivered dose of DPPC for CHF5633 was 2.5 mg per mouse compared with ∼1.75 mg per mouse for poractant alfa, as this preparation contained many other lipid species. Consequently, CHF5633 delivered a greater amount of DPPC to the lungs than did poractant alfa, which may partly explain the higher mean enrichment of surfactant DPPC in BALF at the earliest time point measured from mice given CHF5633 (82%) compared with poractant alfa (53%) expressed relative to total recovered DPPC (endogenous + exogenous). However, these doses of exogenous surfactant are lower than the high amounts of radiolabeled surfactant that have previously been demonstrated to enhance the clearance of exogenous surfactant from mouse lungs (39). Of course, further in vivo and clinical investigations are required in order to draw any conclusions as to whether this prolonged residence of CHF5633 in mouse lungs has any potential to translate into therapeutic benefit for surfactant therapy of neonatal respiratory distress syndrome of preterm infants. We cannot discount the possibility that the prolonged residence of CHF5633 may have been in part due to pooling in the airways and reaching the gas-exchange regions of the lungs more slowly. MS imaging of lavaged lung tissue, however, demonstrated exclusive parenchymal distribution of administered CHF5633 DPPC, with negligible accumulation in the larger airways (results not shown).

This is the first study to combine lipidomic analysis of PC molecular species with the use of a fully stable isotope-labeled DPPC to investigate both the catabolism and subsequent metabolism of exogenous surfactant DPPC. Previous studies have used surfactant preparations containing DPPC labeled with a variety of radioactive and stable isotopes as tracers. Labeled groups in DPPC have included [^3^H]palmitoyl (39), [^14^C]palmitoyl (40), [^3^H]choline (41, 42), [^32^P]phosphate (43), and [16^13^C]palmitoyl (43–47) and have concentrated on the analysis of disaturated PC isolated as the residue after osmium tetroxide oxidation. However, these studies did not provide evidence for the metabolic fate of surfactant PC catabolism. The use of fully labeled [U^13^C]DPPC in this study has several advantages. First, the fully labeled DPPC is extremely unlikely to be resynthesized by any metabolic or recycling route due to the dilution of labeled hydrolytic products with higher concentrations of unlabeled metabolites. Any labeled hydrolytic product that might be recycled into DPPC will generate a PC molecule with a lower molecular mass than [U^13^C]DPPC, which therefore can provide an absolute measure of catabolism of the intact molecule. Second, because all products of [U^13^C]DPPC hydrolysis will also be fully labeled, the extent of metabolic fluxes and metabolism can be quantified. Third, the simultaneous labeling of de novo PC synthesis with methyl-D_9_-choline permits a direct estimate of any effect of exogenous surfactant on the synthesis and turnover of endogenous surfactant PC. The metabolism of [U^13^C]DPPC products into all PC species, and not just DPPC, can be readily determined from the lipidomic analysis.

The analysis of [U^13^C]DPPC enrichment for both labeled poractant alfa and CHF5633, determined by a precursor scan of the [5^13^C]choline head group at *m/z* 189 (P189), showed a high variation of effective surfactant administration even at the earliest time points (supplemental Fig. S3). This was an artifact of the intranasal route chosen for surfactant administration, with the aim of avoiding stresses involved in the intubation of mice. This inherent variation was the reason for analyzing a large number of mice at each time point: to be able to provide a statistical comparison between the catabolism of the two surfactant preparations. The inspection of either mean or maximal values of [U^13^C]DPPC enrichment (Fig. 2 and supplemental Fig. S3) showed a significant initial delay of label decay for both surfactant preparations, which was more prolonged for CHF5633, followed by a phase of more rapid catabolism such that virtually all of the molecules from either surfactant were catabolized by 72 h.

The [U^13^C]DPPC label was incorporated effectively into both surfactant preparations and was metabolized at equilibrium with unlabeled surfactant PC (supplemental Fig. S2). This important conclusion implies that a simple analysis of the fractional concentration of unlabeled DPPC relative to total PC has potential for use for in clinical studies monitoring the clearance of CHF5633, where DPPC is the sole PC species, but not of poractant alfa. We previously demonstrated the feasibility of this approach by analyzing the prolonged duration of excess DPPC in endotracheal aspirates from preterm infants administered the synthetic surfactant Exosurf (48). This would, of course, be at the expense of not obtaining the metabolic analysis provided by using the stable isotope label but might be suitable for a more routine analysis of surfactant clearance in clinical studies.

Previous reports have suggested that exogenous surfactant can enhance the synthesis of endogenous surfactant PC but that this effect depended both on the type of surfactant administered (49) and developmental stage (50). By contrast, the therapeutic equivalent doses of exogenous surfactant delivered in this study had no appreciable effect on the rate of endogenous PC synthesis by the mouse lung, as determined from the incorporation of methyl*-*D_9_-choline (Fig. 3) and compared with previous studies of lung PC synthesis in mice not dosed with exogenous surfactant (13). In the absence of a control group of untreated mice in this study, we made use of the high variation of [U^13^C]DPPC enrichment in BALF (supplemental Fig. S3) to divide the mice post hoc into two groups with higher and lower [U^13^C]DPPC enrichments. The rationale here was that, especially at the earlier time points, this division would generate two equally sized groups at each time point, one of which would have received a higher and the other a lower surfactant dose. Consequently, any effect of increased surfactant dose on endogenous lung PC synthesis would have been expected to modulate the measured incorporation of methyl-D_9_-choline, but no such effect was observed (Fig. 3).

The metabolism of [U^13^C]DPPC from administered surfactants in the lungs was slow and prolonged compared with endogenous PC synthesis and turnover. For instance, the incorporation of methyl-D_9_-choline into lung PC was maximal at the earliest time points (Fig. 4), while that of [5^13^C]choline derived from [U^13^C]DPPC was only maximal between 18 and 24 h and, at least for CHF5633, maintained until 48 h (Fig. 6). Comparable prolonged timescales were evident for the reacylation of [24^13^C]lysoPC16:0 with polyunsaturated fatty acids (Fig. 7) and, especially, for the reincorporation of [16^13^C]palmitate into DPPC (Fig. 9). Presumably, these delayed and sustained incorporations reflect a relatively slow formation of labeled metabolic products destined for reincorporation into lung PC. Furthermore, the transient nature of these metabolic processes in poractant alfa-treated mice compared with CHF5633-treated mice is probably related to the more rapid clearance of this surfactant, and thereby not allowing time for the metabolic products of [U^13^C]DPPC to accumulate in the lungs. Our study, however, provides no direct evidence for the mechanism underlying this enhanced clearance of poractant alfa, but it does indicate that this is not associated with the enhanced accumulation of hydrolysis products in the lungs. The relatively low enrichment values for the recycling of [5^13^C]choline hydrolysis fragments suggests that this is not a major route for exogenous surfactant catabolism in adult mice but nevertheless are sufficient to show no preferential resynthesis of DPPC.

This is the first study to explore in molecular species the various mechanisms and pathways for the recycling of exogenous surfactant delivered to mouse lungs. The overall conclusions are that, while their routes of metabolism are similar, CHF5633 was cleared significantly more slowly from adult mouse lungs than poractant alfa, the rate of active remodeling of DPPC from exogenous surfactant was low, and the synthesis of endogenous surfactant PC was not significantly impaired after the administration of either surfactant. This is consistent with a similar study in rabbits (22) that demonstrated, using radioactively labeled substrates, extensive recycling of surfactant PC by neonatal but not adult animals. It does not, of course, preclude and cannot quantify the extent to which intact surfactant PC is taken up and resecreted by ATII cells without hydrolysis and recycling of its metabolic products, as suggested by studies in rabbit lungs (42, 51). The differences between catabolism and metabolism of CHF5633 and poractant alfa PC are intriguing and may be due to their different phospholipid compositions, but this will require further investigation.

## Supplementary Material

Supplemental Data
