# Supplementary material for: Metabolism of a synthetic compared with a natural therapeutic pulmonary surfactant in adult mice
Source: J Lipid Res. 2018 Aug 14;59(10):1880–92. doi: 10.1194/jlr.M085431 (PMC6168297; doi:10.1194/jlr.M085431)
Supplement: Supplemental Data [file supp_59_10_1880__index.html]

Metabolism of a synthetic compared with a natural therapeutic pulmonary surfactant in the adult mouse. — Metabolism of a synthetic compared with a natural therapeutic pulmonary surfactant in adult mice — Supplemental Data 

# Metabolism of a synthetic compared with a natural therapeutic pulmonary surfactant in adult mice

## Supplemental Data

- Supplemental material (.pdf, 197 KB) - Supplemental material
