# Supplementary material for: Metabolism of a synthetic compared with a natural therapeutic pulmonary surfactant in adult mice
Source: J Lipid Res. 2018 Aug 14;59(10):1880–92. doi: 10.1194/jlr.M085431 (PMC6168297; doi:10.1194/jlr.M085431)
Supplement: Supplemental Data [file 10.1194_M085431_jlr.M085431-1.pdf]

Metabolism of a synthetic compared with a natural therapeutic pulmonary surfactant in the adult mouse.

Jens Madsen[a d], Madhurban H. Panchal[a], Rose-Marie A. Mackay[a], Mercedes Echaide[c], Grielof Koster[a, d, d], Giancarlo Aquino[b], Nicola Pelizzi[b], Jesus Perez-Gil[c], Fabrizio Salomone[b], Howard W. Clark[a,] Anthony D. Postle[a,d]

[a]Child Health, Clinical and Experimental Sciences, Faculty of Medicine, University of Southampton, Southampton, U.K; [b]Chiesi Farmaceutici SpA, Parma, Italy and [c]Dept. of Biochemistry and Molecular Biology, Fac. of Biology and Research Institut "hospital 12 de Octubre", Complutense University, Madrid, Spain; [d]National Institute for Health Research, Biomedical Research Centre, University Hospitals Southampton, Southampton, U.K.

Supplemental material

Corresponding author:

Professor A.D. Postle

Clinical and Experimental Sciences, Faculty of Medicine, Southampton General Hospital, Tremona Road, Southampton, SO16 6YD; Tel: ++44(0)2381206161; e-mail: adp@soton.ac.uk

Running title:

Lung surfactant metabolism in mice

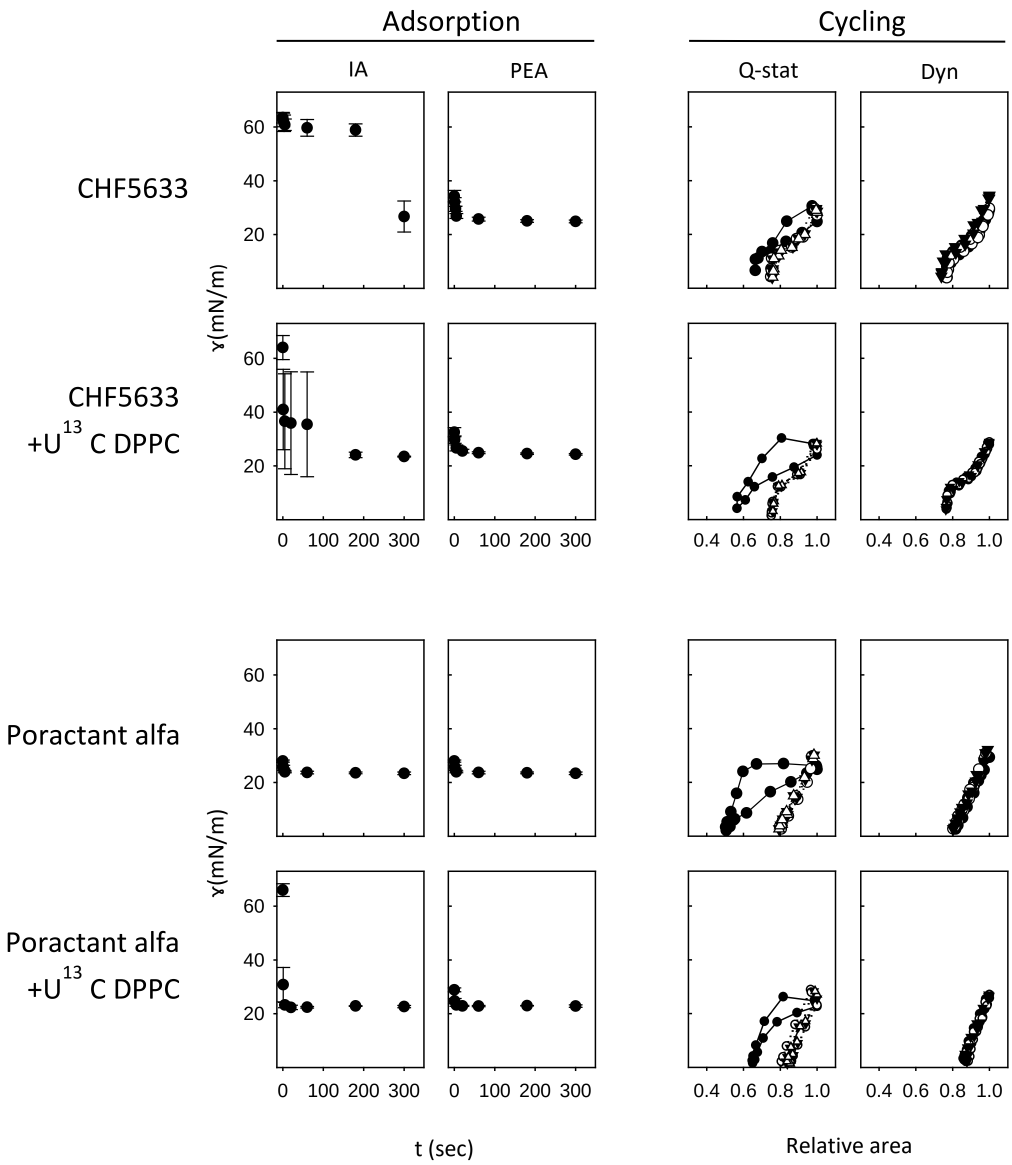

Supplement Figure S1. Comparative functional analysis of films formed by U^{13}C labelled- and non-labelled Poractant alfa and CHF5633 preparations. Performance of labelled and non-labelled preparations of CHF5633 (upper panels) and Poractant alfa (lower panels) surfactant preparations was compared first with respect to the ability to adsorb quickly at the air-water interface of an air bubble (left panels), either initially after injection (initial adsorption, IA) or upon further bubble expansion (post-expansion adsorption, PEA). Plotted in IA and PEA graphs are mean values with standard deviation after averaging three independent experiments. Afterwards, the performance of the formed interfacial films once subjected to compression-expansion cycling are compared in the cycling isotherms (right panels), obtained either at very low rate including discrete steps to allow for

eventual film relaxation (quasi static, Q-stat), or under rapid breathing-like dynamics (20 cycles/min, Dyn). The graphs illustrate representative cycling isotherms after repeating the experiment 3 times. Plotted are the first (black symbols), 3rd and 5th of the Q-stat cycles and the 1st (black symbols), 10th and 20th of the Dyn cycles.

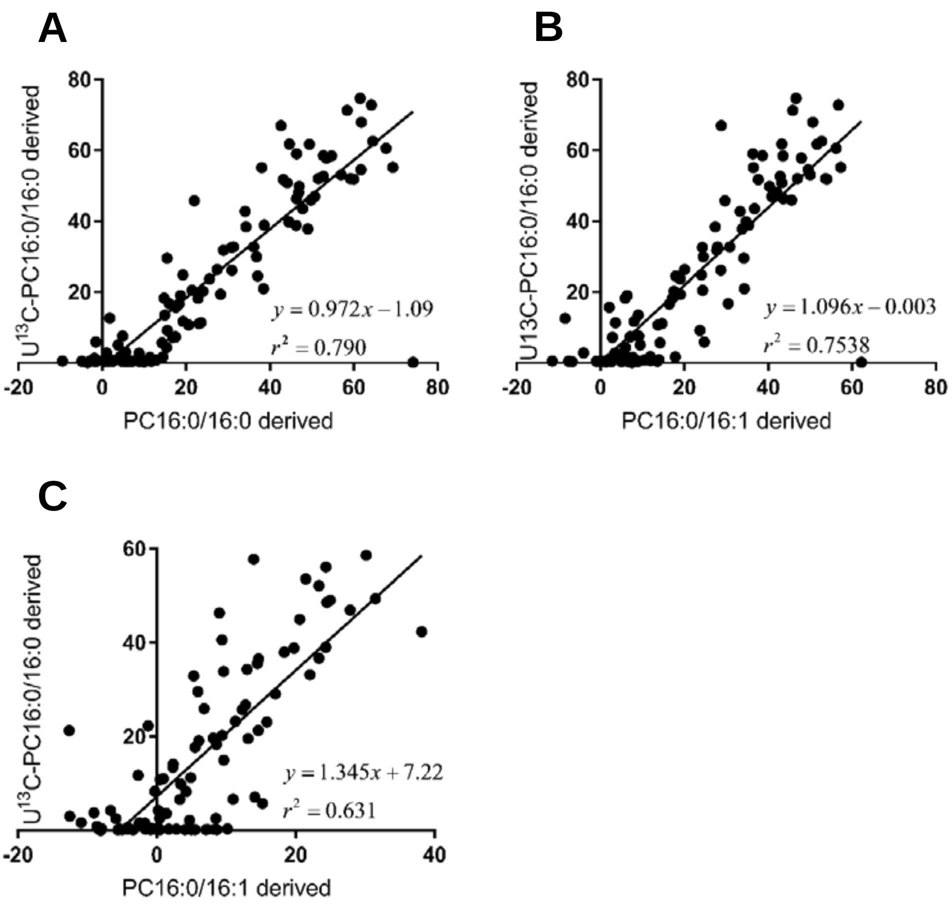

Supplement Figure S2. Exogenous surfactant concentrations (% total DPPC) in BALF from CHF5633 (A, B) and Poractant alfa (C) treated mice. These figures show results from all mice at all time points. Exogenous surfactant concentrations were calculated both by recovery of $U^{13}C$-DPPC (y-axis) and from the differences in composition of unlabelled PC (x-axes) between mouse surfactant and both CHF5633 and Poractant alfa. For panel A, as DPPC is the only PC species in CHF5633, fractional DPPC content above that of mouse surfactant (44.6%) provided a direct measure of exogenous surfactant concentration (x-axis). A comparable concentration could not be made for Poractant alfa as this had a similar DPPC content to mouse surfactant. Mouse surfactant was relatively enriched in PC16:0/16:1 (25.9%) compared with CHF5633 (0%) and Poractant alfa (10.6%) and this provided an alternative approach to quantifying exogenous surfactant concentration in B. CHF5633 and C. Poractant alfa.

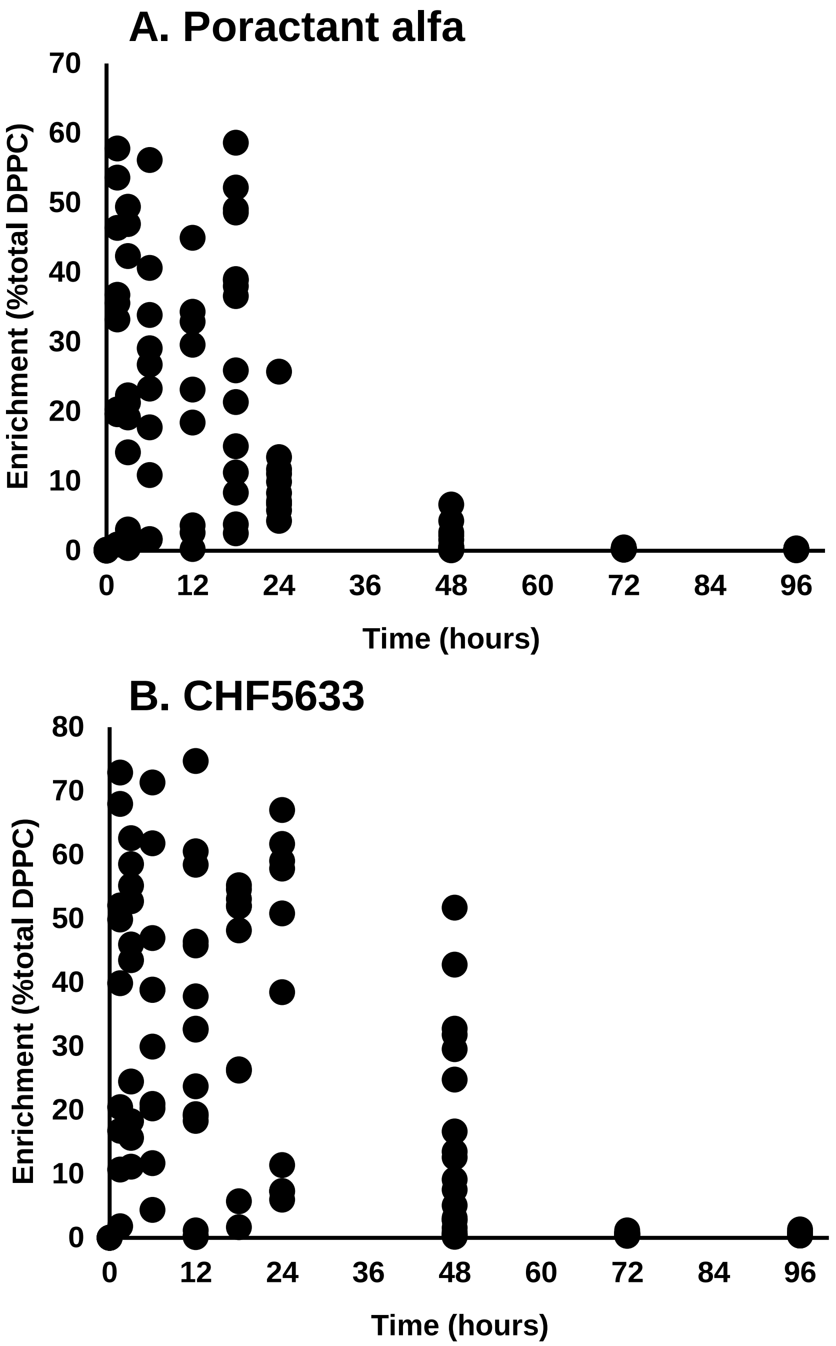

Supplement Figure S3. Enrichment of U13C-DPPC in BALF. Results are shown for individual mice administered either A. Poractant alfa or B. CHF5633. Values have been normalised for the enrichments of the administered surfactant preparations to enable direct comparison between the two groups of mice. Results are expressed relative to total DPPC content (labelled + unlabelled).
